# Mild hypothermia combined with dexmedetomidine reduced brain, lung, and kidney damage in experimental acute focal ischemic stroke

**DOI:** 10.1186/s40635-022-00481-4

**Published:** 2022-12-19

**Authors:** Denise Battaglini, Adriana Lopes da Silva, Nathane Santanna Felix, Gisele Rodrigues, Mariana Alves Antunes, Nazareth Novaes Rocha, Vera Luiza Capelozzi, Marcelo Marcos Morales, Fernanda Ferreira Cruz, Chiara Robba, Pedro Leme Silva, Paolo Pelosi, Patricia Rieken Macedo Rocco

**Affiliations:** 1 Anesthesiology and Critical Care, San Martino Policlinico Hospital, IRCCS for Oncology and Neurosciences, 16132 Genoa, Italy; 2 Department of Medicine, University of Barcelona, 08007 Barcelona, Spain; 3 Laboratory of Pulmonary Investigation, Carlos Chagas Filho Institute of Biophysics, Centro de Ciências da Saúde, Federal University of Rio de Janeiro, Avenida Carlos Chagas Filho, 373, Bloco G-014, Ilha Do Fundão, Rio de Janeiro, RJ 21941-902 Brazil; 4 Department of Physiology and Pharmacology, Biomedical Institute, Fluminense Federal University, Niterói, 24220-900 Brazil; 5 Department of Pathology, University of São Paolo, São Paolo, 05508-060 Brazil; 6 Laboratory of Cellular and Molecular Physiology, Carlos Chagas Filho Institute of Biophysics, Federal University of Rio de Janeiro, Rio de Janeiro, 21941-901 Brazil; 7 Department of Surgical Sciences and Integrated Diagnostics (DISC), University of Genoa, Genoa, Italy; 8 Rio de Janeiro Network On Neuroinflammation, Carlos Chagas Filho Foundation for Supporting Research in the State of Rio de Janeiro (FAPERJ), Rio de Janeiro, Brazil

**Keywords:** Neuroprotection, Hypothermia, Ischemic stroke, Animal model, Kidney

## Abstract

**Background:**

Sedatives and mild hypothermia alone may yield neuroprotective effects in acute ischemic stroke (AIS). However, the impact of this combination is still under investigation. We compared the effects of the combination of mild hypothermia or normothermia with propofol or dexmedetomidine on brain, lung, and kidney in experimental AIS. AIS-induced Wistar rats (*n* = 30) were randomly assigned, after 24 h, to normothermia or mild hypothermia (32–35 °C) with propofol or dexmedetomidine. Histologic injury score and molecular biomarkers were evaluated not only in brain, but also in lung and kidney. Hemodynamics, ventilatory parameters, and carotid Doppler ultrasonography were analyzed for 60 min.

**Results:**

In brain: (1) hypothermia compared to normothermia, regardless of sedative, decreased tumor necrosis factor (TNF)-α expression and histologic injury score; (2) normothermia + dexmedetomidine reduced TNF-α and histologic injury score compared to normothermia + propofol; (3) hypothermia + dexmedetomidine increased zonula occludens-1 expression compared to normothermia + dexmedetomidine. In lungs: (1) hypothermia + propofol compared to normothermia + propofol reduced TNF-α and histologic injury score; (2) hypothermia + dexmedetomidine compared to normothermia + dexmedetomidine reduced histologic injury score. In kidneys: (1) hypothermia + dexmedetomidine compared to normothermia + dexmedetomidine decreased syndecan expression and histologic injury score; (2) hypothermia + dexmedetomidine compared to hypothermia + propofol decreased histologic injury score.

**Conclusions:**

In experimental AIS, the combination of mild hypothermia with dexmedetomidine reduced brain, lung, and kidney damage.

## Background

Stroke is a leading cause of long-term disability, impaired quality of life, and mortality worldwide [1]. Focal ischemic stroke can enhance leukocyte infiltration, increasing the production of toxic metabolites capable of destroying the blood–brain barrier and leading to cell death [2]. Stroke has also been associated with systemic inflammation, which may lead to distal organ damage [2, 3].

Novel therapies have been proposed for neuroprotection in stroke patients. Dexmedetomidine (an α2-adrenergic agonist) and propofol (2,6-diisopropylphenol) seem to decrease the cerebral metabolic rate, without compromising cerebral autoregulation, and exert anti-inflammatory, antioxidant, and anti-apoptotic properties [4, 5]. Mild hypothermia may also induce neuroprotection in experimental studies [6, 7]; however, there are controversies in human clinical trials [8, 9]. Sedatives (such as propofol or dexmedetomidine) and mild hypothermia alone have been shown to present neuroprotective effects and reduce brain damage [10]. However, the impact of this combination is still under investigation in both pre-clinical and clinical settings [11, 12].

This study aimed to compare the effects of the combination of mild hypothermia or normothermia with propofol or dexmedetomidine on brain, lung, and kidney in experimental acute focal ischemic stroke, reproducing an intensive care unit scenario regarding the use of controlled mechanical ventilation, as well as continuous hemodynamic and respiratory monitoring [13].

## Materials and methods

### Study approval

This study was approved by the Ethics Committee for the Use of Animals (CEUA-CCS-116/19) of Federal University of Rio de Janeiro, Brazil. All animals received care in accordance with the “Principles of Laboratory Animal Care”, formulated by the National Society for Medical Research and the “Guide for the Care and Use of Laboratory Animals” from the National Academy of Sciences, USA. Animal experiments were performed in compliance with ARRIVE guidelines^13^.

### Animal preparation and the experimental protocol

Thirty male Wistar rats (body weight, 359–420 g) were anesthetized with xylazine 2.5 mg kg^−1^ and ketamine 75 mg.kg^−1^ intraperitoneally (i.p.), placed in a stereotactic frame with their heads immobilized, and then underwent acute focal ischemic stroke by thermocoagulation of the pial blood vessels that cover the somatosensory, motor and primary sensorimotor cortex [2]. The procedure consists of craniotomy after the skin incision, exposing the left frontoparietal cortex (+ 2 to − 6 mm antero-posterior based on the bregma). Transdural thermocoagulation of blood in the superficial vessels was performed by approaching with a hot probe adjusted to 300 °C [2]. Vessel lesions were evaluated macroscopically through the color changes after 5 min. The incision tissue was sutured, the animals were kept warm under a heating lamp, and oxygen was administered (fraction of inspired oxygen [FiO_2_] of 0.3) during and after the surgical procedure. After recovery from anesthesia, the animals were returned to their cages. The stroke procedure was performed by the same experienced investigator (A.L.S.), enabling the same stroke severity pattern. Tramadol (10 mg kg^−1^) was administered intramuscularly to relieve pain every 8 h for 24 h [2].

After 24 h, animals were anesthetized (sodium thiopental 50 mg kg^−1^, i.p.), and an intravenous (i.v.) catheter (Jelco 24G; Becton, Dickinson and Company, USA) was inserted into the tail vein, and continuous anesthesia was initiated (T0) [14]. After reaching an adequate depth of anesthesia, animals were tracheostomized, and another catheter (18G; Arrow International, USA) placed in the right internal carotid artery for arterial blood gas analysis (ABL80 FLEX; Radiometer Medical, Denmark). Mean arterial pressure (MAP) and rectal temperature were monitored continuously (Networked Multiparameter Veterinary Monitor Life Window 6000 V; Digicare Animal Health, Florida, USA). The core temperature was measured continuously through the right internal carotid artery (CCA) using a PiCCO catheter (PV2013L07-A; Pulsion Medical Systems, Feldkirchen, Germany). Neuromuscular blockade was achieved with pancuronium bromide (1 mg kg^−1^ i.v.). Animals were then mechanically ventilated (Servo-I; MAQUET, Solna, Sweden) in volume-controlled mode, with V_T_ = 6 ml kg^−1^, respiratory rate = 80 breaths min^−1^, FiO_2_ = 0.4, and positive end-expiratory pressure (PEEP) = 3 cmH_2_O. Airflow and airway pressure were recorded continuously using a computer running custom-made software (LabVIEW; National Instruments, Austin, TX, USA) [15, 16]. Tidal volume, respiratory system plateau pressure (Pplat, RS) and driving pressure (ΔP, RS) were measured [2, 15] (MATLAB, version R2007a; MathWorks Inc, Natick, MA, USA).

**Fig. 1:**
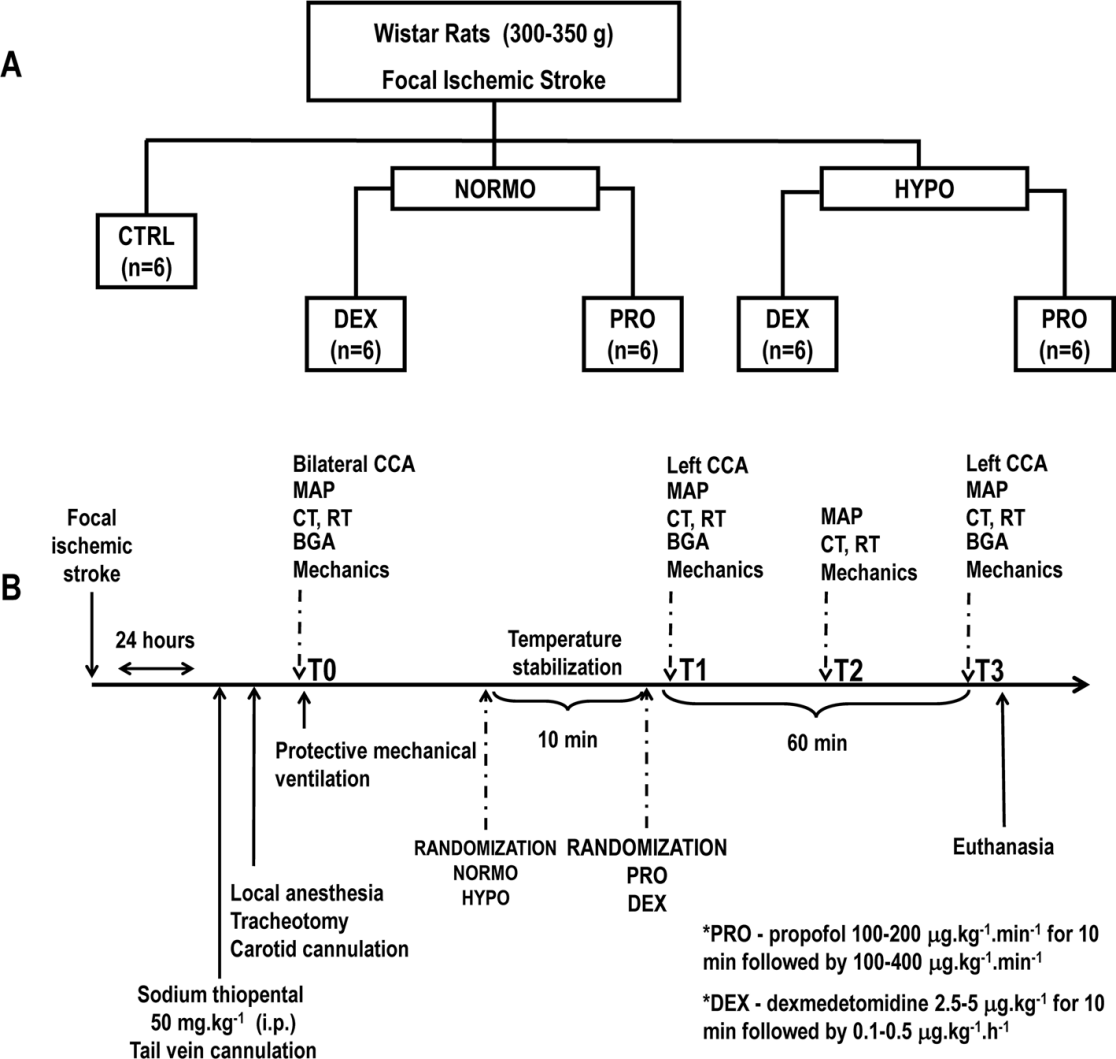
Schematic flowchart of the study design (top) and timeline representation of the experimental protocol (bottom). **A** Animals were randomly assigned to normothermia (NORMO) or mild hypothermia (HYPO) combined with propofol (PRO) or dexmedetomidine (DEX): NORMO + PRO, HYPO + PRO, NORMO + DEX, HYPO + DEX groups (*n* = 6, each). **B** Study timeline from the time of stroke induction to the end of the experimental protocol. *BGA* blood gas analysis, *cCT* continuous core temperature, *CD* carotidal Doppler, *cMAP* continuous mean arterial pressure, *cRT* continuous rectal temperature, *T0* baseline, T1, T2, and T3 = 15, 30, and 60 min after temperature stabilization

After hemodynamic and respiratory stabilization, the animals were randomly assigned to normothermia or mild hypothermia combined with propofol or dexmedetomidine: NORMO + PRO, HYPO + PRO, NORMO + DEX, HYPO + DEX groups (*n* = 6, each) (Fig. 1A). After AIS induction, animals from the control (CTRL) group were evaluated under normothermia. They were then anesthetized with sodium thiopental (50 mg kg^−1^) and protectively mechanically ventilated. CTRL animals were then compared to the randomized groups for molecular biomarkers. To maintain the core temperature between 37.8 °C and 38.3 °C [17] (normothermia), animals were warmed using a thermostatically controlled heating pad (EFF 421, Insight; Brazil) and an infrared light positioned 30 cm from the body of the rat to reach a normothermic condition. The hypothermia protocol was induced by spraying 75% alcohol on the rats’ body until a stable temperature compatible with mild hypothermia (between 34 °C and 35 °C) was reached [10, 18, 19]. Temperature stabilization was achieved after 10 min (T1) and the animals then received dexmedetomidine (Precedex; Laboratories Abbott do Brasil Ltda., São Paulo, SP, Brazil) with a bolus of 5 μg kg^−1^ i.v. for 10 min and then an infusion of 0.1–0.5 μg kg^−1^ h^−1^ i.v. for 50 min or propofol (Propovan; Laboratories Cristália from Brazil Ltda., Itapira, São Paulo, SP, Brazil) via an initial infusion of 100–200 μg kg^−1^ min^−1^ i.v. for 10 min and then infusion of 100–400 μg kg^−1^ min^−1^ i.v. for 50 min. Both infusion rates were based on previous studies that showed no adverse hemodynamic effects in rats during infusion for 60 min [2, 14]. Animals were kept at normothermia or hypothermia until the end of the experiment (T3). At T3, all animals received heparin (1000 IU) intravenously into the tail vein and euthanized by an overdose of sodium thiopental (150 mg kg^−1^). Brain, lungs, and kidneys were carefully removed for histology and molecular biology analysis. Histology of the heart was also performed. Data on hemodynamics, gas exchange, and respiratory system mechanics were collected at T0, T1, T2, and T3 (Fig. 1B). We opted to focus on our primary hypothesis, which was to compare the effects of the combination of mild hypothermia or normothermia with propofol or dexmedetomidine on brain, lung, and kidney in experimental acute ischemic stroke. No *sham* animals were included in the current study. All data regarding *sham* animals were previously produced by our laboratory and published elsewhere [2]. The resulting reduction of the number of groups led to a more reliable statistical analysis.

### Carotid Doppler ultrasonography

Phased-arrayed ultrasonography was performed using a miniaturized probe and a color-coded and duplex Doppler ultrasonographic system (Samsung UGEO HM70 system; São Paulo, SP, Brazil) with an 8–13 MHz linear transducer. The assessments were performed with the rats in the supine position. The probe was directed parallel to the blood stream, and the angle was maintained at < 20°. All ultrasonic readings were evaluated and appraised by two investigators with experience in carotid sonography (N.R. and D.B.). Carotid Doppler ultrasonography [20] of both CCAs was performed at T0 and from the left CCA at T1 and T3 (Fig. 1B). The peak systolic velocity (PSV) (cm s^−1^), end-diastolic velocity (EDV) (cm s^−1^), mean flow velocity (mFV) (extrapolated from the following formula: mFV = SFV + 2 × EDV/3), where SFV is the systolic flow velocity [21], pressure gradient (PG = 4V^2^) [22–24], and the pulsatility index (PI) (PI = (PSV − EDV)/MV) were calculated.

### Histologic injury score

Tissues from the brain (perilesional area [penumbra] located in the right hemisphere of the ischemic lesion), left lung, right kidney, and heart were extracted, fixed in 4% paraformaldehyde for 24 h, and embedded in paraffin. Tissue section (5 μm thick) were cut (Leica RM2135 Leica Biosystem, São Paulo, Brazil) and stained with hematoxylin and eosin. Eight fields of view per section were studied through a light microscope (Olympus BX51; Olympus Latin America, Brazil), at × 25, × 100, × 400 and × 1000 magnification. Perilesional brain, lung, kidney, and heart injury scores were quantified using a scale to represent the severity of apoptosis, edema, inflammation, and necrosis (0, no effect; 4, maximum severity). The extension of each feature was graded as 0 for no appearance and 4 for complete involvement. Final scores were calculated as the product of severity and extent of each feature, ranging from 0 to 16. The cumulated brain, lung, kidney, and heart injury score ranged from 0 to 64 [25]. All histological analyses were performed by two investigators (G.R. and V.L.C.), blinded to the group allocation. The scores of each expert were combined to yield a final score by arithmetic averaging [25]. The value of kappa was 0.86.

### Molecular biomarkers in brain, lung, and kidney tissues

A small specimen of the brain tissue (about 3–4 mm) was collected for the molecular biomarker analysis from the rectangular perilesional area (*penumbra*), located medially to the right hemisphere of ischemic lesion. Additionally, the right lung, and left kidney tissues were extracted, quickly frozen and stored at − 80 °C. RT-PCR was performed to measure molecular biomarkers associated with inflammation (tumor necrosis factor [TNF]-α, interleukin [IL]-1β), endothelial cell damage (intercellular adhesion molecule-1 [ICAM-1]) and tight junction protein zonula occludens-1 (ZO-1) (a biomarker that establishes a link between the transmembrane protein occludin and the actin cytoskeleton) in perilesional brain tissue. The expression of proinflammatory markers (TNF-α and IL-6), ICAM-1 and a molecular biomarker associated with inflammation and fibrosis (E-selectin) were evaluated in central slices of right lung tissue. The expression of kidney injury molecule (KIM)-1 (a biological biomarker associated with kidney injury), IL-6, syndecan (a heparan sulfate proteoglycan associated with tubular epithelial cell damage), and ZO-1 was evaluated in the kidney tissue. Total RNA was extracted from brain, lung, and kidney frozen tissues with the ReliaPrep RNA Tissue Miniprep System (Promega Corporation, Fitchburg, Wisconsin, USA), following the manufacturer’s recommendations. RNA concentration was measured by spectrophotometry in a Nanodrop ND-2000 system (Thermo Fisher Scientific, Wilmington, DE, USA). First-strand cDNA was synthesized from total RNA using the High-Capacity cDNA Reverse Transcription Kit (Thermo Fisher, Waltham, MA, USA). Relative mRNA levels were measured with a BRYT Green system (Promega, Fitchburg, WI, USA) using PCR Mastercycler ep Realplex Eppendorf (Eppendorf, Hamburg, Germany). Samples were measured in triplicate. The primers are shown in Table 1.

**Table 1:** Forward and reverse oligonucleotide sequences of target gene primers

Gene	Primer	Primer sequences (5′–3′)
Brain		
TNF-α	Forward	CAGCCGATTTGCCATTTCATAC
	Reverse	GGCTCTGAGGAGTAGACGATAA
IL-1β	Forward	CTATGTCTTGCCCGTGGAG
	Reverse	CATCATCCCACGAGTCACA
ICAM-1	Forward	CTTCCGACTAGGGTCCTGAA
	Reverse	CTTCAGAGGCAGGAAACAGG
ZO-1	Forward	CACCACAGACATCCAACCAG
	Reverse	CACCAACCACTCTCCCTTGT
Lung		
IL-6	Forward	CTCCGCAAGAGACTTCCAG
	Reverse	CTCCTCTCCGGACTTGTGA
TNF-α	Forward	CAGCCGATTTGCCATTTCATAC
	Reverse	GGCTCTGAGGAGTAGACGATAA
E-selectin	Forward	TGCCAAGAACAGGAATACCC
	Reverse	CTCCCAGGATTTGAGGAACA
ICAM-1	Forward	CTTCCGACTAGGGTCCTGAA
	Reverse	CTTCAGAGGCAGGAAACAGG
Kidney		
KIM-1	Forward	GAAGAAAACAATGGATCAAGGGAT
	Reverse	GGAGTGGAAATGGCTCTAATGAAC
ZO-1	Forward	CACCACAGACATCCAACCAG
	Reverse	CACCAACCACTCTCCCTTGT
Syndecan	Forward	GTTCCGCTGGTTTTGTTGTTT
	Reverse	GATGAAGGCTGTTCCCAGGTA
IL-6	Forward	CTCCGCAAGAGACTTCCAG
	Reverse	CTCCTCTCCGGACTTGTGA
* 36B4*	Forward	GGATCACTCAGGAGCAGGAG
	Reverse	CTTGGCACTCAAGAGGAAGG

*TNF* tumor necrosis factor, *ICAM* intercellular adhesion molecule, *ZO-1* zonula occludens-1, *KIM* kidney injury molecule-1, *IL-6* interleukin-6, *IL-1β* interleukin-1 beta, *36B4* acidic ribosomal phosphoprotein P0

For each sample, the expression of each gene was normalized to the acidic ribosomal phosphoprotein P0 (*36B4*) housekeeping gene and expressed as the fold change relative to CTRL, using the 2^−ΔΔCt^ method, where ΔCt = Ct (target gene) − Ct (reference gene) [26]. Blinded analyses were carried out by one investigator (M.A.A.).

### Statistical analysis

The sample size was calculated based on pilot studies to allow detection of differences between mild hypothermia and normothermia in TNF-α expression in brain- injured rats, regardless of the sedative used. The number of animals per group (*n* = 6) was calculated based on a power of 80%, α = 5%, two-tailed, and effect size (*d*) of 1.37 (G*Power 3.1.9.2; University of Düsseldorf, Düsseldorf, Germany). The primary outcome was TNF-α expression in brain tissue, and the secondary outcomes were brain, lung, and kidney histology and molecular biology. Data were tested for normality using the Kolmogorov–Smirnov test with Lilliefors’ correction, and the Levene median test was used to evaluate homogeneity of variances. Differences in the parameters among the groups and over time were compared using the two-way ANOVA test followed by the Holm–Šídák post hoc test or two-way ANOVA for non-parametric data with Tukey’s multiple comparison. Significance was established at *p* < 0.05. For histologic and molecular biology variables, Mann–Whitney test followed by the Bonferroni multiple comparisons were done. *p* value (*p* < 0.0125) was adjusted for four comparisons: hypothermia + dexmedetomidine, normothermia + dexmedetomidine, hypothermia + propofol, and normothermia + propofol. Parametric data were expressed as means ± SD and non-parametric data as medians (interquartile range). All statistical analyses were performed using SPSS version 23.0 and GraphPad version 8.2.1.

## Results

### Hemodynamics and respiratory function during the experiments

MAP remained higher than 80 mmHg in all groups. In T1, MAP was significant higher (*p* = 0.02) in hypothermia + propofol compared to hypothermia + dexmedetomidine.

In the right common carotid artery, at T0, PSV and PG were higher in hypothermia + dexmedetomidine compared to normothermia + dexmedetomidine (PSV *p* = 0.009 and PG *p* = 0.002) (Table 2). In the left common carotid artery, at T0, PSV, EDV and MV were higher in hypothermia + dexmedetomidine compared to normothermia + dexmedetomidine (PSV *p* = 0.039, EDV *p* = 0.018, and MV *p* = 0.022, respectively). At T0, EDV was higher in hypothermia + dexmedetomidine compared to hypothermia + propofol (EDV *p* = 0.036). At T1 and T3, PSV and MV were higher in hypothermia + propofol compared to normothermia + propofol (PSV T1 *p* = 0.041; PSV T3 *p* = 0.029; MV T1, *p* = 0.040; MV T3 *p* = 0.031). At T1 and T3, MV was higher in hypothermia + dexmedetomidine compared to normothermia + dexmedetomidine (MV T1 *p* = 0.042; MV T3 *p* = 0.042). PG and PI did not differ among the groups at any time (Table 2).

**Table 2:** Ultrasound of common carotid arteries

Ultrasound parameter	Group		T0	T1	T3	Group effect	Time effect	Group × time effect
Left carotid								
PSV (cm/s)	NORMO	DEX	56.42 (45.21–82.28)	64.01 (55.38–95.68)	81.78 (61.15–100.70)	*p* = 0.005	*p* < 0.001	*p* = 0.429
	HYPO		80.18 (70.66–90.35)	91.03 (81.68–95.84)	96.27 (94.47–104.90)	T0 NORMO-DEX vs HYPO-DEX, *p* = 0.039 TI NORMO-PRO vs HYPO-PRO, *p* = 0.041 T3 NORMO-PRO vs HYPO-PRO, *p* = 0.029		
	NORMO	PRO	61.25 (57.60–70.38)	66.39 (61.14–73.64)	73.93 (65.11–75.91)			
	HYPO		79.92 (64.60–91.56)	87.49 (80.64–90.90)	90.43 (85.73–100.20)			
EDV (cm/s)	NORMO	DEX	3.67 (2.63–6.11)	5.36 (3.84–6.77)	6.29 (4.99–7.50)	*p* = 0.019	*p* < 0.001	*p* = 0.556
	HYPO		7.74 (5.49–10.18)	8.93 (6.01–9.59)	9.93 (6.54–10.09)	T0 NORMO-DEX vs HYPO-DEX, *p* = 0.018 T0 HYPO-DEX vs HYPO-PRO, *p* = 0.036		
	NORMO	PRO	3.92 (2.90–4.71)	3.97 (3.01–5.93)	5.62 (4.56–6.76)			
	HYPO		4.39 (3.71–6.59)	5.83 (4.22–7.35)	7.22 (5.15–7.78)			
MV (cm/s)	NORMO	DEX	59.31 (47.38–85.28)	67.24 (58.29–101.10)	85.98 (64.52–105.00)	*p* = 0.003	*p* < 0.001	*p* = 0.425
	HYPO		84.21 (76.71–95.30)	97.33 (85.77–101.60)	102.90 (101.30–109.20)	T0 NORMO-DEX vs HYPO-DEX, *p* = 0.022 T1 NORMO-PRO vs HYPO-PRO, *p* = 0.040 TI NORMO-DEX vs HYPO-DEX, *p* = 0.042 T3 NORMO-PRO vs HYPO-PRO, *p* = 0.031 T3 NORMO-DEX vs HYPO-DEX, *p* = 0.042		
	NORMO	PRO	64.32 (59.62–73.17)	69.34 (63.33–77.38)	69.34 (63.33–77.38)			
	HYPO		82.85 (67.90–95.13)	90.88 (85.54–94.47)	95.24 (90.13–102.70)			
PG (mmHg)	NORMO	DEX	1.38 (0.93–1.93)	1.77 (1.09–2.79)	2.72 (1.55–3.32)	*p* = 0.122	*p* < 0.001	*p* = 0.636
	HYPO		2.08 (1.81–3.07)	2.68 (1.91–3.23)	3.60 (3.00–3.89)			
	NORMO	PRO	1.40 (1.26–1.71)	1.82 (1.57–2.24)	2.27 (1.82–2.88)			
	HYPO		1.39 (0.77–1.92)	1.87 (1.28–2.60)	2.45 (1.89–3.38)			
PI	NORMO	DEX	2.46 (2.30–2.78)	2.59 (2.43–3.61)	2.95 (2.70–3.86)	*p* = 0.213	*p* < 0.001	*p* = 0.696
	HYPO		2.93 (2.39–4.13)	3.19 (2.91–4.22)	3.63 (3.46–4.33)			
	NORMO	PRO	2.74 (2.49–3.16)	2.98 (2.70–3.31)	3.06 (2.96–3.35)			
	HYPO		2.81 (2.66–2.98)	2.98 (2.91–3.49)	3.62 (2.99–3.84)			
Right carotid								
PSV (cm/s)	NORMO	DEX	49.93 (44.70–64.81)	–	–	T0 HYPO-DEX vs NORMO-DEX, *p* = 0.009		
	HYPO		70.30 (64.86–83.90)	–	–			
	NORMO	PRO	53.85 (39.73–60.27)	–	–			
	HYPO		55.42 (48.35–64.20)	–	–			
EDV (cm/s)	NORMO	DEX	5.39 (3.76–6.21)	–	–	–		
	HYPO		8.26 (5.68–10.74)	–	–			
	NORMO	PRO	4.87 (4.05–5.28)	–	–			
	HYPO		3.66 (2.75–6.05)	–	–			
MV (cm/s)	NORMO	DEX	52.67 (48.01–68.79)	–	–	–		
	HYPO		77.13 (69.81–87.74)	–	–			
	NORMO	PRO	57.48 (42.88–63.14)	–	–			
	HYPO		57.87 (50.30–68.86)	–	–			
PG (mmHg)	NORMO	DEX	1.98 (1.86–2.14)	–	–	T0 HYPO-DEX vs NORMO-DEX, *p* = 0.002		
	HYPO		3.12 (2.78–4.43)	–	–			
	NORMO	PRO	2.12 (1.89–2.84)	–	–			
	HYPO		1.83 (1.65–2.69)	–	–			
PI	NORMO	DEX	2.91 (2.30–3.37)	–	–	–		
	HYPO		3.40 (2.95–4.14)	–	–			
	NORMO	PRO	2.98 (2.27–3.67)	–	–			
	HYPO		2.87 (2.48–3.19)	–	–			

Values are presented as medians (interquartile range) of 6 animals/group. *PSV* peak systolic velocity, *EDV* end-diastolic velocity, *MV* mean velocity, *PG* pressure gradient, *PI* Pulsatility Index of left and right common carotid arteries (CCA) at T0, T1, and T3. Two-way ANOVA test with Tukey’s multiple comparison was used for the left carotid over time, considering significance at *p* < 0.05; Mann–Whitney test was adopted for the right carotid at baseline, assuming significance at *p* < 0.0125. PSV in the right carotid at T0 was higher in mild hypothermia + dexmedetomidine (HYPO-DEX) than normothermia + dexmedetomidine (NORMO-DEX), *p* = 0.009. No differences at T0 in EDV and PI in the right CCA were found among the groups. At T0, PG was significantly higher in HYPO-DEX than NORMO-DEX in the right CCA

At T0 and T1, Pplat, RS was higher in hypothermia + propofol compared to normothermia + propofol (Pplat, RS T0 *p* = 0.026; T1 *p* = 0.008) (Table 3). No significant differences were found in V_T_ and ΔP, RS (Table 3) as well as gas exchange between groups (Table 4).

**Table 3:** Respiratory mechanics

Parameter	Group	T0	T1	T2	T3	Group effect	Time effect	Group vs. time effect
V_T_ (mL/kg)						*p* = 0.246	*p* = 0.132	*p* = 0.174
NORMO	DEX	6.67 (5.91–7.12)	6.67 (6.21–7.12)	6.90 (6.55–7.97)	6.67 (6.19–7.59)			
HYPO		7.35 (7.04–7.94)	7.73 (7.10–8.65)	7.48 (7.10–8.47)	7.94 (7.59–8.62)			
NORMO	PRO	6.84 (6.39–7.76)	6.76 (6.32–7.77)	7.00 (6.62–7.83)	7.13 (6.60–7.88)			
HYPO		7.73 (6.31–9.46)	7.51 (6.31–8.14)	8.34 (7.29–9.14)	7.50 (7.05–7.69)			
Pplat, RS (cmH_2_O)						*p* = 0.003	*p* = 0.020	*p* = 0.961
NORMO	DEX	14.10 (11.45–15.65)	14.20 (11.40–15.55)	16.10 (1.93–16.30)	14.80 (12.45–16.80)	T0 HYPO-PRO vs NORMO-PRO, *p* = 0.026 T1 HYPO-PRO vs NORMO-PRO, *p* = 0.008		
HYPO		14.30 (12.90–16.72)	16.15 (14.39–16.73)	15.90 (13.55–17.10)	16.45 (15.23–16.80)			
NORMO	PRO	9.25 (8.80–11.19)	11.03 (8.65–11.93)	11.50 (9.90–12.05)	11.10 (9.28–13.30)			
HYPO		13.84 (12.20–15.43)	15.24 (13.10–16.80)	14.55 (13.05–16.30)	14.40 (11.70–16.80)			
ΔP, RS (cmH_2_O)						*p* = 0.017	*p* < 0.001	*p* = 0.476
NORMO	DEX	11.00 (8.00–12.10)	10.50 (8.80–12.00)	12.80 (9.18–13.30)	11.50 (9.90–14.40)			
HYPO		10.75 (9.20–12.93)	12.68 (11.35–13.08)	12.55 (9.85–14.51)	12.70 (11.50–16.76)			
NORMO	PRO	6.25 (5.78–7.59)	8.08 (6.03–9.03)	9.15 (8.38–9.65)	9.45 (8.95–10.78)			
HYPO		9.99 (8.63–12.45)	11.44 (9.53–13.22)	10.87 (9.41–13.29)	10.65 (8.38–13.23)			

Data are expressed as medians (interquartile range) of 6 animals/group. Two-way ANOVA for non-parametric data was used with Tukey’s multiple comparison, *p* was considered statistically significant for values < 0.05. At T3, Mann–Whitney test was used with Bonferroni comparison, considering *p* < 0.0125 as significant, but no significant differences were found. *HYPO* mild hypothermia, *NORMO* normothermia, *PRO* propofol, *DEX* dexmedetomidine, *V*_*T*_ tidal volume, *Pplat* plateau pressure, *RS* respiratory system, *ΔP* driving pressure

**Table 4:** Gas exchange

Parameter	Group		T0	T1	T3	Group effect	Time effect	Group vs. time effect
pHa	NORMO	DEX	7.54 (7.51–7.57)	7.47 (7.40–7.49)	7.43 (7.40–7.45)	*p* = 0.408	*p* < 0.001	*p* = 0.036
	HYPO		7.50 (7.46–7.55)	7.47 (7.38–7.49)	7.41 (7.27–7.46)			
	NORMO	PRO	7.46 (7.40–7.52)	7.46 (7.36–7.49)	7.41 (7.35–7.50)			
	HYPO		7.51 (7.47–7.53)	7.49 (7.44–7.54)	7.46 (7.39–7.48)			
PaO_2_/FiO_2_	NORMO	DEX	237.50 (168.60–488.00)	381.30 (275.00–446.90)	317.50 (192.50–445.60)	*p* = 0.808	*p* = 0.144	*p* = 0.893
	HYPO		301.30 (171.30–401.90)	428.80 (261.30–461.30)	423.80 (224.40–461.30)			
	NORMO	PRO	303.50 (177.50–438.10)	385.00 (335.00–445.50)	403.80 (384.40–451.30)			
	HYPO		397.50 (190.00–473.10)	406.30 (203.80–489.90)	381.3 (187.50–450.00)			
PaCO_2_ (mmHg)	NORMO	DEX	30.35 (26.40–35.48)	31.45 (29.68–36.18)	29.80 (28.35–31.55)	*p* = 0.118	*p* = 0.383	*p* = 0.108
	HYPO		34.75 (32.05–38.13)	37.45 (29.10–52.00)	38.85 (33.05–47.93)			
	NORMO	PRO	38.25 (32.55–41.60)	32.15 (29.35–40.70)	36.80 (27.33–48.95)			
	HYPO		34.65 (31.95–36.63)	27.60 (26.70–33.10)	36.85 (28.80–39.53)			
HCO_3_^−^ (mEq/L)	NORMO	DEX	24.80 (22.78–30.23)	23.00 (21.05–24.23)	18.80 (18.13–20.98)	*p* = 0.418	*p* < 0.001	*p* = 0.181
	HYPO		26.50 (24.98–28.48)	25.60 (22.38–30.53)	23.40 (18.15–26.15)			
	NORMO	PRO	24.35 (23.23–28.98)	22.35 (20.18–24.68)	23.75 (19.03–26.03)			
	HYPO		27.40 (23.48–30.13)	22.35 (21.25–24.75)	23.30 (21.85–25.35)			

Data are expressed as median (interquartile range). *NORMO* normothermia, *HYPO* hypothermia, *DEX* dexmedetomidine, *PRO* propofol, *PaO*_*2*_ arterial partial pressure of oxygen, *FiO*_*2*_ fraction of inspired oxygen, *PaCO*_*2*_ arterial partial pressure of carbon dioxide, *HCO3*^*−*^ bicarbonate

### Perilesional brain tissue

**Fig. 2:**
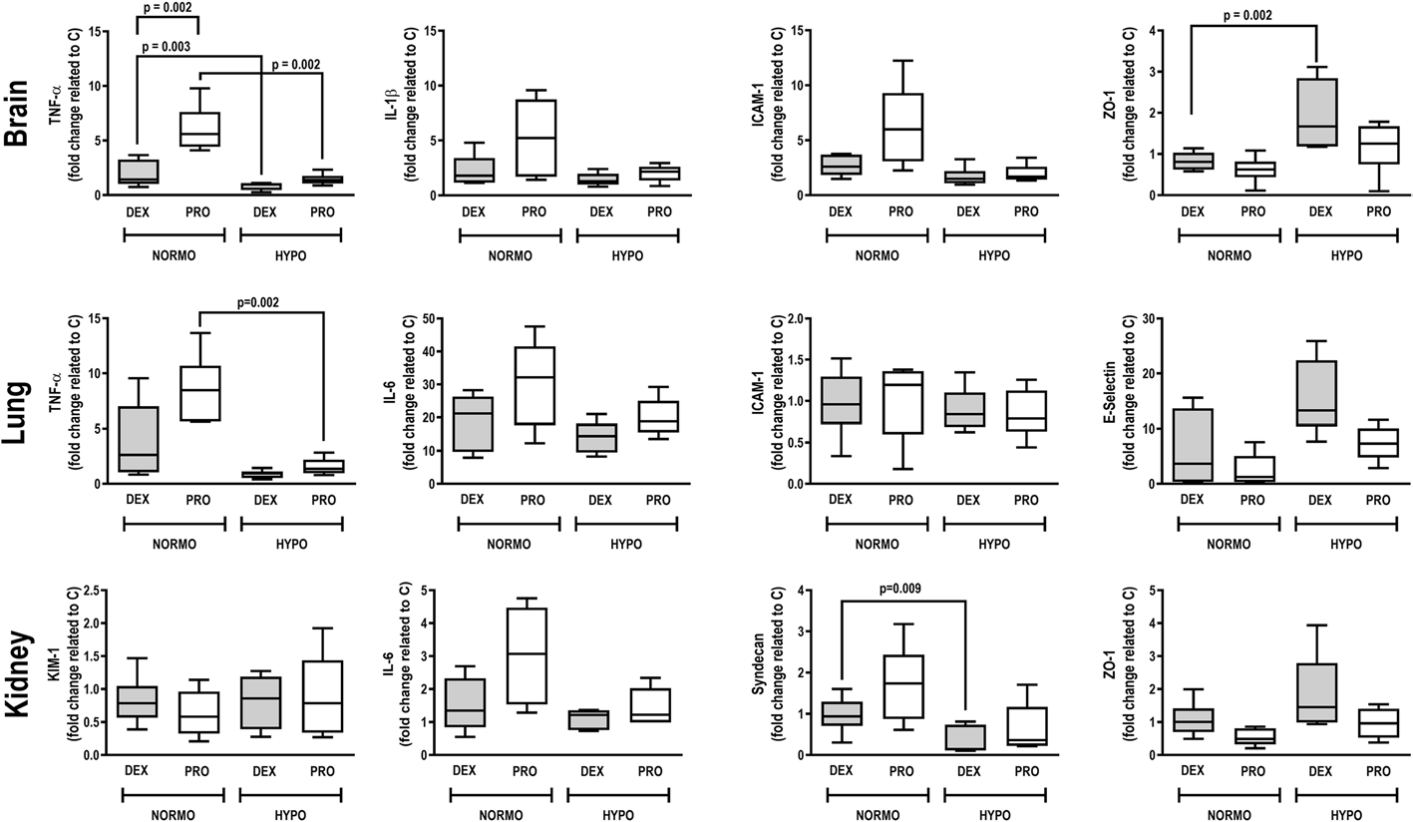
Real-time polymerase chain reaction analysis of proinflammatory biological markers in the brain perilesional, lung, and kidney tissues. Relative gene expression was calculated as the ratio of average gene expression levels compared with the reference gene (36B4) and expressed as fold change relative to controls (C). In the brain perilesional tissue: interleukin-6 (IL-6), IL-1, intercellular adhesion molecule (ICAM), and zonula occludens-1 (ZO-1); in the lung tissue: IL-6, tumor necrosis factor-α, E-selectin, and ICAM; in the kidney tissue: IL-6, kidney injury molecule-1 (KIM1), ZO-1, and syndecan. Data are presented as box plots of medians and interquartile ranges with 6 animals in each group. Statistical significance was considered for *p* < 0.0125. *DEX* dexmedetomidine, *HYPO* hypothermia, *NORMO* normothermia, *PRO* propofol

The mRNA expression of molecular biomarkers in the perilesional brain tissue is reported in Fig. 2. TNF-α expression was lower in normothermia + dexmedetomidine versus normothermia + propofol (*p* = 0.002); and in hypothermia + dexmedetomidine versus normothermia + dexmedetomidine and normothermia + propofol (*p* = 0.003). ZO-1 expression was higher in hypothermia + dexmedetomidine compared to normothermia + dexmedetomidine (*p* = 0.002).

**Fig. 3:**
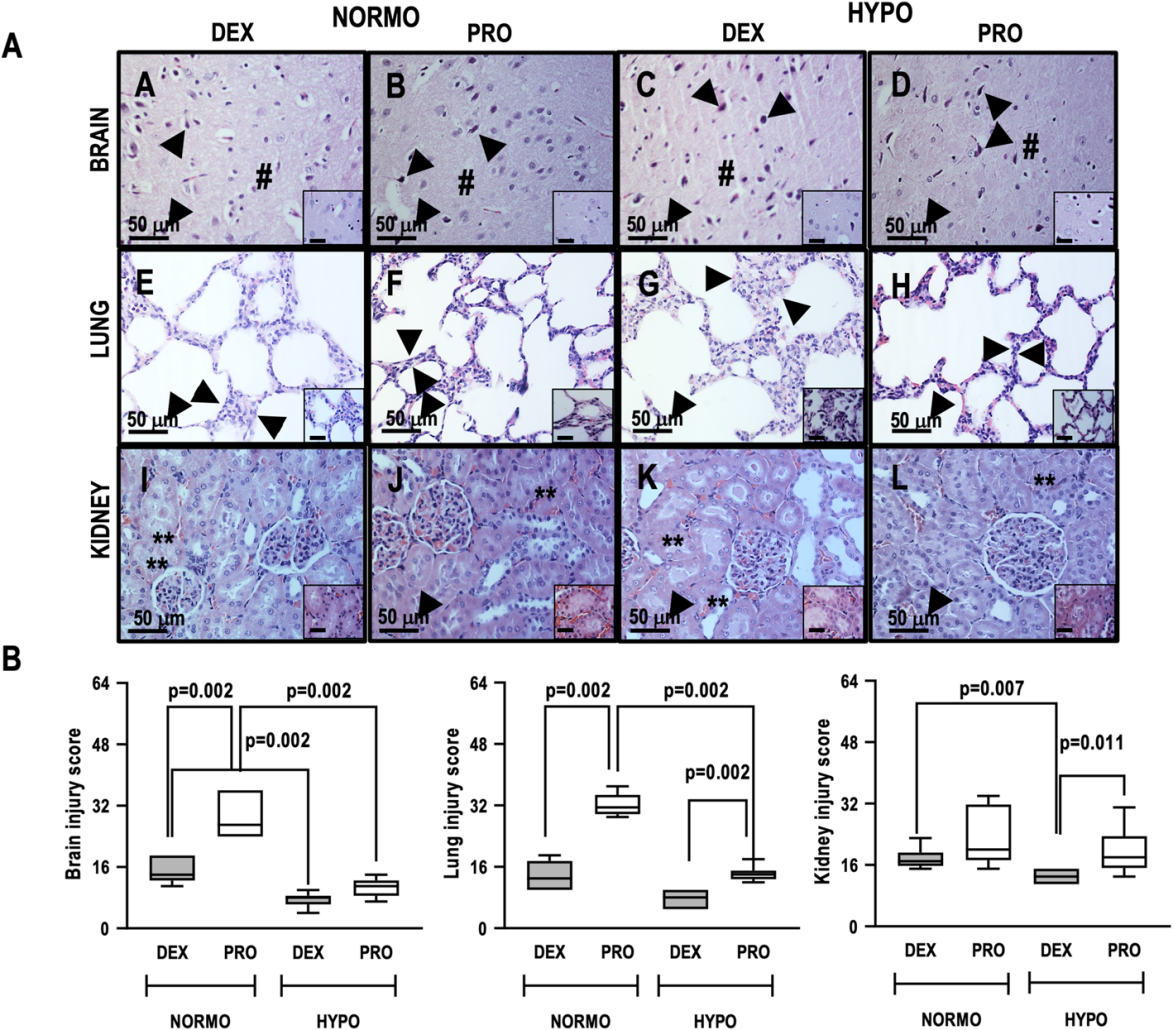
**A** Representative photomicrographs (light microscopy) of brain, lung, and kidney stained with hematoxylin–eosin stain in normothermia propofol (NORMO + PRO), normothermia dexmedetomidine (NORMO + DEX), hypothermia propofol (HYPO + PRO), and hypothermia dexmedetomidine (HYPO + DEX) rats. Decrease in cortex neurons pyroptosis and neuropil edema (**A** arrowheads and hash, inset) were observed in the HYPO + DEX and HYPO + PRO groups. Decrease in inflammatory thickening of the alveolar septa (**E** double arrowheads, inset) and renal tubular necrosis (**I** double asterisks, inset) in the HYPO + DEX and HYPO + PRO groups; more prominent in the NORMO + DEX group. The marked decrease in pyroptosis in cortical neurons (**C** arrowheads, inset), inflammatory alveolar septa (**G** double arrowheads, inset), and renal tubular necrosis (**K** double asterisks, inset) in HYPO + DEX and HYPO + PRO rats (**D**–**L**). Magnification × 400; inset × 1000. **B:** Brain, lung and kidney injury score. Boxes show the interquartile (25–75%) range, whiskers encompass the range (minimum to maximum), and horizontal lines represent median values of six animals/group. *DEX* dexmedetomidine, *HYPO* hypothermia, *NORMO* normothermia, *PRO* propofol. Histologic injury score in brain, lung and kidney was calculated by multiplying the severity and extent of organ injury (minimum score = 0 and maximum score = 16) and the total was calculated as the sum of each score for apoptosis, edema, inflammation, and necrosis (minimum score = 0 to maximum score = 64)

Photomicrographs of perilesional area in each group are shown in Fig. 3A. The brain histologic injury score, which includes the extent and severity of apoptosis, edema, inflammation, and necrosis, is presented in Table 5 and Fig. 3B. The brain injury score was lower in normothermia + dexmedetomidine and hypothermia + propofol versus normothermia + propofol (*p* = 0.002, both); and in hypothermia + dexmedetomidine versus normothermia + dexmedetomidine (*p* = 0.002).

**Table 5:** Damage scores in perilesional brain, lung, and kidney tissues

	NORMO		HYPO		Group effect
	DEX	PRO	DEX	PRO	
Brain					
Brain injury score	(12.50–19.00)	27.00 (24.00–36.00)	8.00 (6.25–8.50)	11.00 (8.50–12.50)	NORMO-PRO vs. NORMO-DEX *p* = 0.002 NORMO-PRO vs. HYPO-PRO *p* = 0.002 NORMO-DEX vs. HYPO-DEX *p* = 0.002
Apoptosis	4.00 (4.00–6.00)	10.50 (8.25–12.00)	2.00 (1.75–2.50)	2.00 (2.00–4.00)	NORMO-PRO vs. NORMO-DEX *p* = 0.007 NORMO-PRO vs. HYPO-PRO *p* = 0.002 NORMO-DEX vs. HYPO-DEX *p* = 0.011
Edema	4.00 (4.00–9.00)	9.00 (8.25–12.00)	3.00 (2.00–4.00)	4.00 (2.75–4.50)	NORMO-PRO vs. HYPO-PRO *p* = 0.004
Inflammation	5.00 (3.00–6.00)	9.00 (8.25–12.00)	2.00 (1.00–4.00)	4.00 (3.75–4.50)	NORMO-PRO vs. NORMO-DEX *p* = 0.009 NORMO-PRO vs. HYPO-PRO *p* = 0.004
Necrosis	0.00 (0.00–0.00)	0.00 (0.00–0.00)	0.00 (0.00–0.00)	0.00 (0.00–0.00)	–
Lung					
Lung injury score	13.00 (10.00–17.50)	31.50 (29.75–34.75)	8.00 (5.00–10.00)	14.00 (12.75–15.00)	NORMO-PRO vs. NORMO-DEX *p* = 0.002 NORMO-PRO vs. HYPO-PRO *p* = 0.002 HYPO-DEX vs. HYPO-PRO *p* = 0.002
Apoptosis	4.00 (4.00–5.25)	10.50 (8.75–16.00)	2.00 (2.00–4.00)	4.00 (3.75–6.00)	NORMO-PRO vs. NORMO-DEX *p* = 0.009 NORMO-PRO vs. HYPO-PRO *p* = 0.002
Edema	4.00 (2.00–5.25)	9.00 (7.50–10.75)	2.00 (2.00–4.00)	6.00 (4.00–6.00)	NORMO-PRO vs. HYPO-PRO *p* = 0.011
Inflammation	4.00 (4.00–6.00)	12.00 (9.00–12.00)	2.00 (1.00–4.00)	4.00 (4.00–4.50)	NORMO-PRO vs. NORMO-DEX *p* = 0.002 NORMO-PRO vs. HYPO-PRO *p* = 0.002
Necrosis	0.00 (0.00–0.00)	0.00 (0.00–0.00)	0.00 (0.00–0.00)	0.00 (0.00–0.00)	–
Kidney					
Kidney injury score	17.00 (15.75–19.25)	20.00 (17.25–31.75)	13.00 (11.00–15.00)	18.00 (15.25–23.50)	NORMO-DEX vs. HYPO-DEX *p* = 0.007 HYPO-DEX vs. HYPO-PRO *p* = 0.011
Apoptosis	5.00 (4.00–5.00)	5.00 (4.00–9.00)	4.00 (4.00–4.00)	4.00 (3.25–5.25)	–
Edema	4.00 (4.00–4.00)	4.00 (2.00–6.00)	2.00 (2.00–4.00)	4.00 (4.00–6.00)	–
Inflammation	4.00 (2.00–4.00)	4.00 (3.50–6.00)	1.50 (1.00–4.00)	4.00 (3.50–5.25)	–
Necrosis	4.00 (3.75–6.00)	8.00 (4.00–12.00)	4.00 (3.75–4.00)	4.00 (4.00–9.00)	–

Data are expressed as medians (interquartile range). Mann–Whitney test with Bonferroni multiple comparison between groups was adopted. Statistical significance was considered for *p* < 0.0125. Points of severity and extent varied between 0 (no severity/extent) and 4 (maximum severity/extent). Histology injury scores were calculated by multiplying the severity and extent of brain injury (minimum score = 0 and maximum score = 16) and was calculated as the sum of each score for apoptosis, edema, inflammation, and necrosis (minimum score = 0 to maximum score = 64). *NORMO* normothermia, *HYPO* hypothermia, *DEX* dexmedetomidine, *PRO* propofol

### Distal organs: lung, kidney, and heart

In lung, the gene expression of TNF-α was significantly lower in hypothermia + propofol compared to normothermia + propofol (*p* = 0.002) (Fig. 2). The expression of IL-6, ICAM-1, and E-selectin did not differ between the groups. In kidney, the expression of syndecan was lower in hypothermia + dexmedetomidine than normothermia + dexmedetomidine (*p* = 0.009), and no significant differences were observed in the expression of KIM-1, IL-6, and ZO-1.

**Fig. 4:**
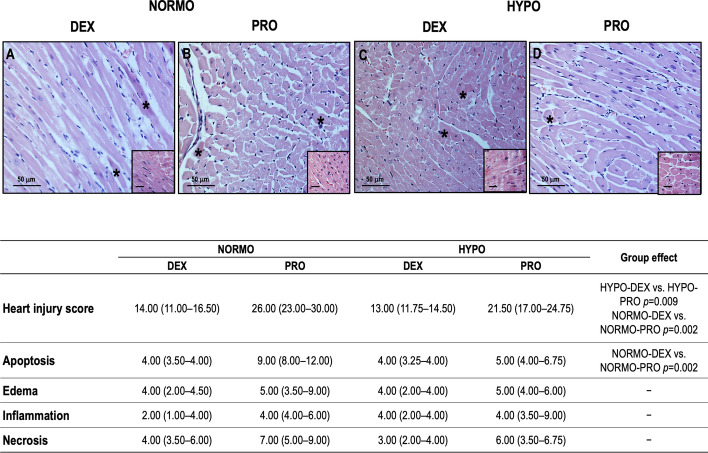
Upper panel. Histology of the heart visualized by hematoxylin–eosin staining in normothermia (NORMO), hypothermia (HYPO), propofol (PRO), dexmedetomidine (DEX) rats. Hyalin necrosis of heart fibers (**A**–**D** asterisks, inset). Magnification × 400; inset × 1000. Lower panel. Data are expressed as median (interquartile range quartiles). Mann–Whitney test with Bonferroni multiple comparison between groups was adopted. A *p* level < 0.0125 was considered statistically significant. Points for severity and extent varied between 0 (no severity/extent) to 4 (maximum severity/extent). The heart injury score was calculated by multiplying the severity and extent of injury (minimum score = 0 and maximum score = 16) and was calculated by the sum of each score for apoptosis, edema, inflammation, and necrosis (minimum score = 0 to maximum score = 64). Magnification × 400; inset × 1000

Figure 3A shows the photomicrographs of lung and kidney parenchyma in all groups. The lung histologic injury score was significantly lower in normothermia + dexmedetomidine than normothermia + propofol or hypothermia + propofol (*p* = 0.002, both), as well as in hypothermia + dexmedetomidine compared to hypothermia + propofol (*p* = 0.002) (Table 5, Fig. 3B). The kidney histologic injury score was significantly lower in hypothermia + dexmedetomidine than normothermia + dexmedetomidine (*p* = 0.007) and hypothermia + dexmedetomidine compared to hypothermia + propofol (*p* = 0.011) (Table 5, Fig. 3B). The degree of lung and kidney apoptosis, edema, and inflammation affected this final histologic injury score. In kidney, necrosis also affected the final histologic injury score. The heart histologic injury score was lower in hypothermia + dexmedetomidine compared to hypothermia + propofol (*p* = 0.009) as well as hypothermia + propofol compared to normothermia + propofol (*p* = 0.002). The degrees of heart apoptosis, edema, inflammation, and necrosis are presented in Fig. 4.

## Discussion

In this model of focal ischemic stroke, normothermia and mild hypothermia interact differently with dexmedetomidine or propofol to reduce brain, lung, and kidney damage. We found that the combination of mild hypothermia with dexmedetomidine decreased inflammation and histologic injury score in the brain, as well as in lung and kidney. Thus, the combination of mild hypothermia or normothermia and sedatives may have different effects on brain and peripheral organs.

The present study has some strengths, including the use of a rat model that presents cerebral vasculature and physiology similar to that of humans [27]; and the analysis of the effects combining different target temperatures (mild hypothermia and normothermia) with different sedatives (propofol and dexmedetomidine) on morphology and molecular biology of brain and distal organs [28]. To date, no study has compared different target temperatures combined with different sedatives focusing not only on brain injury but also evaluating beneficial effects on lung, kidney, and heart in acute focal ischemic stroke.

### Perilesional brain tissue

The present study showed that, in the brain, under normothermia, dexmedetomidine, compared with propofol, resulted in a lower brain histologic injury score and inflammation (TNF-α). Our results are in agreement with the literature [29, 30]. Indeed, dexmedetomidine, when compared with saline, decreased inflammation [31], brain water content and damage to the blood–brain barrier, thus improving neurologic function in rat model [32]. In an animal model, propofol was comparable to dexmedetomidine to minimize brain injury, since it reduced oxidative stress, apoptosis [33], microglia-mediated proinflammatory cytokines [34], as well as increased expression of heme-oxygenase-1 in ischemic penumbra and core [35]. Furthermore, propofol potentiates neurologic recovery [36] and neurobehavioral outcome [37, 38] through a decrease in myeloperoxidases, nuclear factor (NF)-κB, cyclooxygenase (COX)-2, and TNF-α [39], which reduces cerebral edema and protects the blood–brain barrier. In pre-clinical studies, dexmedetomidine increased anti-inflammatory and neuroprotective effects more than propofol [40, 41], and this is in accordance to our findings; however, in the clinical setting, dexmedetomidine and propofol appeared equally effective on brain recovery and outcome [42, 43]. These differences may be explained based on the time these sedatives were administered, the type and degree of brain damage, as well as the parameters used to evaluate the efficacy of dexmedetomidine and propofol.

Previous experimental studies show that mild hypothermia reduces infarct size, improves functional outcome, and reduces brain inflammation and apoptosis [44, 45]. In this line, in the current acute ischemic stroke model, expression of TNF-α in the brain and the histologic injury score were significantly reduced in mild hypothermia compared with normothermia using either propofol or dexmedetomidine. The reduced brain injury and decreased inflammation resulting from mild hypothermia with propofol seems to be explained by mechanisms associated with different pathways [12]. Dexmedetomidine associated with hypothermia reduces brain damage, improves neurological outcome, and increases the survival rate of the hippocampal CA1 neurons, compared to saline [46]. The increase in expression of ZO-1 in mild hypothermia + dexmedetomidine compared to normothermia + dexmedetomidine may be attributed to hypothermia alleviating neurocyte apoptosis [47].

### Peripheral organs: lung, kidney, and heart

In the current model of acute ischemic stroke, mild hypothermia + propofol compared to normothermia + propofol reduced TNF-α in lung tissue. In previous experimental studies, under normothermia, 1 h of propofol infusion reduced the expression of proinflammatory mediators [48, 49] and lung injury [50]. Dexmedetomidine but not propofol reduced lung injury in experimental acute ischemic stroke [51]. The reduction of lung damage in hypothermia + dexmedetomidine has been previous observed in a model of acute lung injury [52]. As previously demonstrated in small animal models, other organs can benefit from hypothermia and dexmedetomidine [53–55]. Hypothermia + dexmedetomidine compared to hypothermia + propofol decreased kidney damage, which may be associated with reduced inflammation and oxidative stress [53] through the inhibition of different pathways [54, 55]. In the heart, the histologic injury score was lower with dexmedetomidine than propofol regardless of the temperature. Dexmedetomidine attenuates cell damage and apoptosis in H9c2 cardiomyocytes [56] and inhibits pyroptosis in myocardial ischemia–reperfusion injury in rats [57]. Propofol also reduced cardiac injury via inhibition of intrinsic apoptotic pathways [58, 59].

### Additional findings

In mild hypothermia, we found that carotid flow velocities were increased with both dexmedetomidine and propofol, and mean arterial pressure was lower with dexmedetomidine. In agreement with our results, mild hypothermia increases cerebral blood flow [60], but can be associated with cardiovascular instability [61].

In our study, mild hypothermia with propofol, but not dexmedetomidine, increased Pplat, RS, but lung inflammation was lower with propofol and mild hypothermia. Differently from what reported in a previous study, under normothermia, propofol compared with pentobarbital sodium, reduces airway resistance as well as alveolar collapse in rat models [48]. These contrasting findings need further investigation.

### Limitations

This study has some limitations that should be addressed. First, our model cannot reproduce the complex clinical scenario of human patients. Indeed, the craniectomy to induce focal ischemic stroke, which allows the permanent occlusion of the target vessel by thermocoagulation, has good reproducibility concerning the infarct size, but cannot be comparable to real clinical scenario [62]. Second, our findings are limited to a relatively short observation time (60 min) to hinder lung damage associated with prolonged invasive mechanical ventilation. Third, the location and intensity of damage to the brain, lungs, kidneys and heart and the response to the target temperature, sedatives and different therapeutic conditions might be influenced by the species and size of the animals. The time frame for quantification of gene expression of 1 h, although sufficient to produce changes in mRNA expression, might not have significantly modified protein levels. Fourth, the rats were never awakened to test neurobehavioral response, but were euthanized to collect organs and investigate biomarkers associated with inflammation and histopathological findings**.** Fifth, the organ injury evaluation based on hematoxylin and eosin technique is relatively unspecific given that the degree of apoptosis and necrosis was not confirmed by any specific staining. Despite these limitations, this is a step forward for clinical testing of combined mild hypothermia and sedatives in acute ischemic stroke patients.

## Conclusions

In the current model of acute focal ischemic stroke, the combination of mild hypothermia with dexmedetomidine reduced brain, lung, and kidney damage.

## Data Availability

Datasets generated during and/or analyzed during the current study are available from the corresponding author on reasonable request. https://github.com/DenBatt/DenBatt-2022-.git.

## Abbreviations

CTRL: Control
CCA: Right internal carotid artery
DEX: Dexmedetomidine
EDV: End-diastolic velocity
FiO2: Fraction of inspired oxygen
HYPO: Hypothermia
ICAM: Intracellular adhesion molecule
IL: Interleukin
KIM: Kidney injury molecule
MAP: Mean arterial pressure
mFV: Mean flow velocity
MV: Mean velocity
NORMO: Normothermia
PEEP: Positive end-expiratory pressure
PG: Pressure gradient
PI: Pulsatility index
Pplat: Plateau pressure
PRO: Propofol
PSV: Peak systolic velocity
TNF: Tumor necrosis factor
ZO: Zonula occludens
